# Fungal Bioactive Anthraquinones and Analogues

**DOI:** 10.3390/toxins12110714

**Published:** 2020-11-12

**Authors:** Marco Masi, Antonio Evidente

**Affiliations:** Dipartimento di Scienze Chimiche, Università di Napoli Federico II, Complesso Universitario Monte Sant’Angelo, Via Cintia 4, 80126 Napoli, Italy; marco.masi@unina.it

**Keywords:** anthraquinones, natural analogues, fungi, biological activity

## Abstract

This review, covering the literature from 1966 to the present (2020), describes naturally occurring fungal bioactive anthraquinones and analogues biosynthesized by the acetate route and concerning several different functionalized carbon skeletons. Hydrocarbons, lipids, sterols, esters, fatty acids, derivatives of amino acids, and aromatic compounds are metabolites belonging to other different classes of natural compounds and are generated by the same biosynthetic route. All of them are produced by plant, microorganisms, and marine organisms. The biological activities of anthraquinones and analogues comprise phytotoxic, antibacterial, antiviral, anticancer, antitumor, algicide, antifungal, enzyme inhibiting, immunostimulant, antiplatelet aggregation, cytotoxic, and antiplasmodium activities. The review also covers some practical industrial applications of anthraquinones.

## 1. Introduction

**Figure 1:**
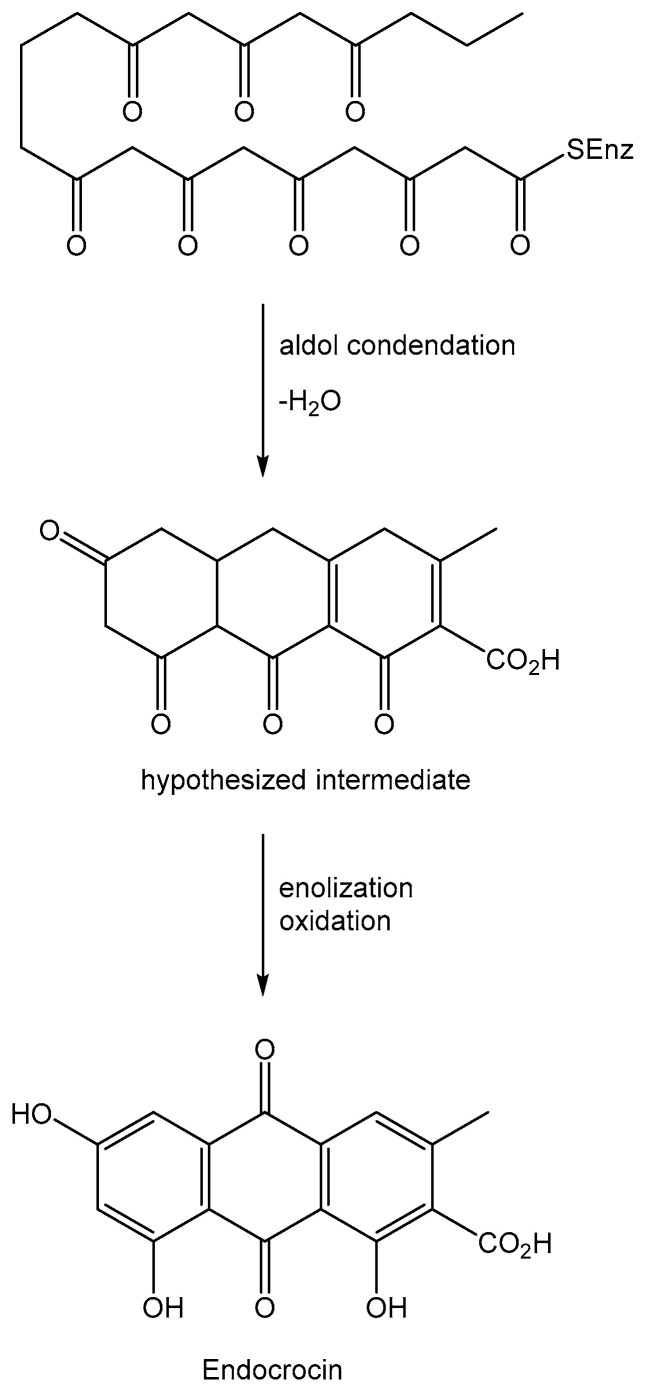
Biosynthesis of the anthraquinone carbon skeleton.

Anthraquinones are a group of natural compounds with a plethora of biological activities and potential practical applications. Most of them are produced by plant and micro-organisms among the living organisms [1,2]. They are acetate-derivative metabolites biosynthesized starting from a polyketide containing eight C2 units, which generates in turn with three aldol type condensations the carbon skeleton of anthraquinones except for the two carbonyl oxygens of the central ring. The latter are introduced by successive steps with an oxidation process. One example of this kind of biosynthesis is reported in Figure 1 for endocrin, a fungal anthraquinone produced by several *Penicillium* and *Aspergillus* species [3].

Among secondary metabolites anthraquinones are the most investigated natural products for their mechanism of action [4]. Plants, microorganisms, lichens, and algae are producers of metabolites possessing diverse biological activities such as phytotoxic, antibacterial, antiviral, anticancer, antitumor, algicide, antifungal, enzyme inhibiting, immunostimulant, antiplatelet aggregation, cytotoxic and antiplasmodium activities. Anthraquinones are frequently reported among the plethora of different classes of natural compounds as alkaloids, hydrocarbons, lipids, sterols, esters, fatty acids, derivatives of amino acids, terpenoids, and aromatic compounds [5,6,7]. The activity of several hydroxyl- and amino-anthraquinones cannot be exploited due to their weak solubility in water. Thus, some of them are converted into water-soluble analogues by biotransformation [8]. Anthraquinones have also industrial application as natural dyes substituting synthetic chemicals in formulation to avoid undesired collateral effects [9].

Some previous reviews have described fungal anthraquinones such as that published by Gessler at al. (2013) [10], limited to 12 natural anthraquinones, and those on anthraquinones specifically produced by derived marine fungi [11]; also among them the treatment of the anthraquinones synthesized by a single fungal species such as *Phoma* was reported [12].

This review describes an advanced overview on anthraquinones, and related analogues grouped for the first time according to their natural sources. In particular, in addition to the isolation from fungal sources and their chemical characterization, their potential applications in different fields such as agriculture, medicine and the dyes industry are considered on the basis of their biological activities.

The first section chronologically describes the fungal anthraquinones starting from 1966 to the present day focusing on their sources, structures, and biological activities. The second section treats the industrial application of anthraquinones in a different field essentially as natural dyes. This part is focused on the comparison between natural and synthetic anthraquinone based dyes, their chemical derivatization and classification, and the advanced methods used in the treatment of the relative industrial wastewater to avoid severe negative environmental pollution. Finally, the main points described are summarized in the conclusion.

## 2. Fungal Anthraquinones and Analogues

**Figure 2:**
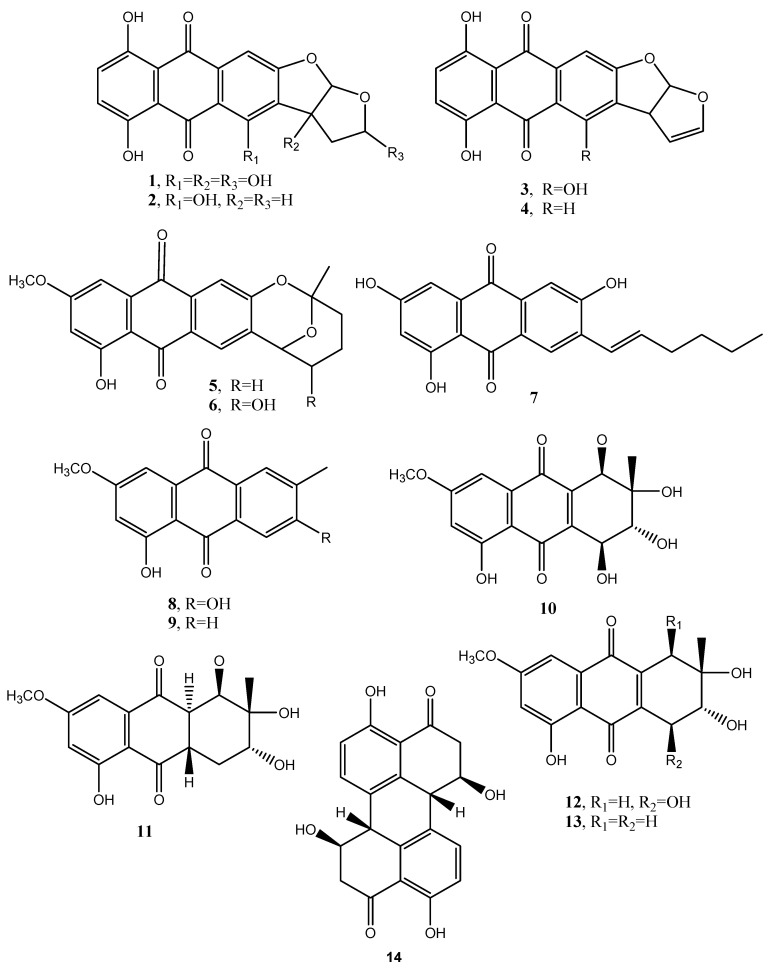
Bioactive anthraquinones and analogues produced by *Dothistroma pini, Aspergillus versicolor, Alternaria porri, Alternaria solani, Alternaria cucumerina, Alternaria bataticola, Diaporthe angelicae* and *Stemphyfium botryosum*.

Dothistromin (**1**, Figure 2, Table 1) was isolated as the main phytotoxin produced by *Dothistroma pini* (Hulbary), a pathogen inducing necrotic disease characterized by the formation of red bands on the infected needles of *Pinus radiata* and other pines [13]. The same fungus also produced six other anthraquinones: bisdeoxydothistromin; bisdeoxydehydrodothistromin; 6-deoxyversicolorin C; averufin; nidurufin; averythrin (**6**–**11**, Figure 2, Table 1). No biological activity was reported for **2**–**7** [14]. More recently, averythrin was also isolated from the marine derived fungus *Aspergillus versivolor* [15].

Macrosporin and 6-methylxanthopurpurin 3-methyl ether (**8** and **9**, Figure 2, Table 1) are two anthraquinones produced by *Alternaria bataticola*, the causal agent of a black spot of sweet potato [16]. Compound **8** was also isolated from other fungi of the same genus as *A. porri*, *A. solani* and *A. cucumerina* while **9** was isolated also from *A. solani* [16]. Then macrosporin was isolated together with another two anthraquinones, named altersolanols A and J (**10** and **11**, Figure 2, Table 1), as well as nectriapyrone, an α-pyrone, from the culture filtrates of *Diaporthe angelicae* (anamorph *Phomopsis foeniculi*), the causal agent of serious disease on fennel (*Foeniculum vulgare*) in Bulgaria. These four metabolites were tested on detached tomato leaves and only nectriapyrone and altersolanols A and J showed a modulate phytotoxicity while macrosporin was not toxic [17].

**Figure 3:**
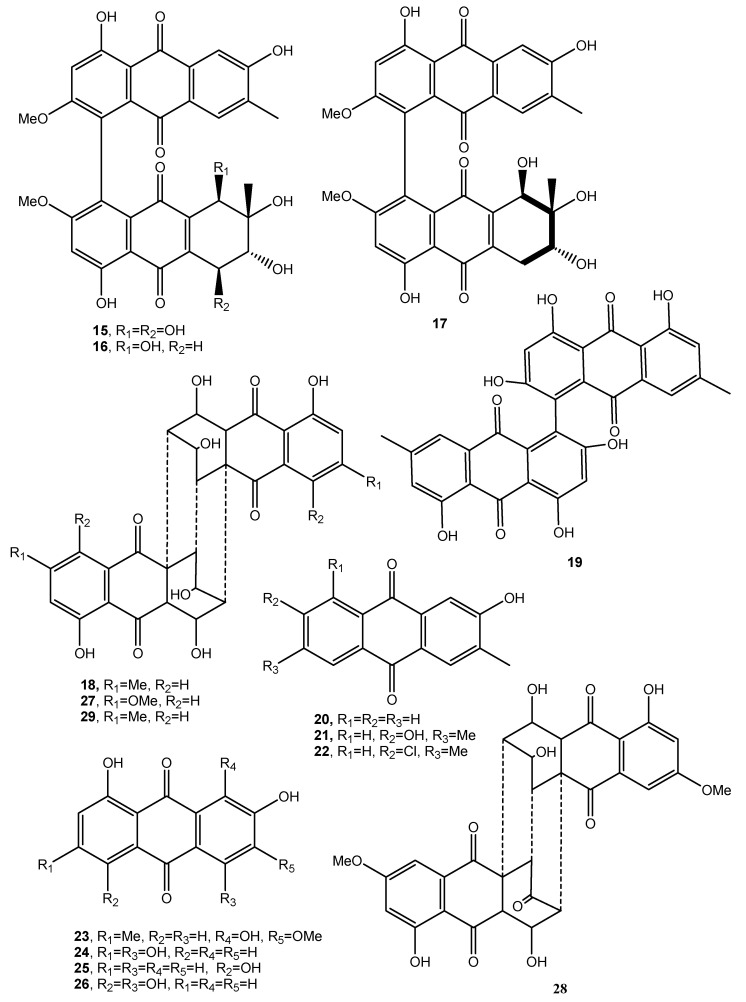
Bioactive anthraquinones and analogues produced by *Stemphyfium botryosum, Cryphonectria parasitica, Hormonema dematioides, Microsporum* sp., *Aspergillus claucus, Pyrenophora tritici-repentis, Gliocladium* sp. *T31, Aspergillus fumigatus, Drechslera teres, Drechslera graminea, Drechslera tritici-repentis, Drechslera phlei, Drechslera dictyoides, Drechslera avenae, Bipolaris sorokinana* and *Cytospora* CR200.

Stemphylin, is a phytotoxin (**12**, Figure 2, Table 1) produced by *Stemphyfium botryosum*, a fungal pathogen inducing a destructive disease in lettuce. The first structure of compound **12** was wrongly assigned by Barash et al. (1975 and 1978) [18,19], and then corrected when it was isolated from the same fungus together with two other phytotoxic anthraquinones, the above cited macrosporin and dactylariol (**13**, Figure 2, Table 1) [20]. The latter compound (**13**) showed anti Adenosine TriPhosphate (ATP) catabolism in Erlich I ascite tumor cells while stemphylin showed a weak antitumor activity on the treated animal at a dose of 40 mg/kg [20]. *Stemphylium botryosum*, inducing leaf spot disease on beet plants, also synthesized macrosporin and dactylariol (**8** and **13**), together with other anthraquinones identified as stemphyrperylenol (**14**, Figure 2), alteroporiol, and stemphynols A and B (**15**–**17**, Figure 3, Table 1). The phytotoxicity of all the metabolites (**8**, **13**–**17**) was tested on lettuce and beet evaluating the seeds elongation. Compound **13** was the most active while compound **14** exhibited a moderate inhibition while the other ones were inactive [21].

*Cryphonectria* (*Endothia*) *parasitica* (Murr.) Barr, the causal agent of chestnut (*Castanea sativa*) canker disease and other species produces diaporthin, a phytotoxic benzopyranone pigment, together with phytotoxic anthraquinones. The hundreds of fungal strains were grouped into virulent, intermediate and hypervirulent and produced, respectively, diaporthin, rugulosin and skirin (**18** and **19**, Figure 3, Table 1), crysophanol (**20**, Figure 3, Table 1) and emodin (**21**, Figure 3, Table 1) [22]. Virulent and hypovirulent strains of *C. parasitica* also produced a main polysaccharide identified as pullulan (a polymer constituted of α-1,4- and α-1,6-glucan) and a minor fraction which gave phytotoxicity on both the host plant and tomato (*Lycopersicon escultem* L.) leaves. One component of this minor fraction was identified as a galactan and consisted of the repeat unit: [→6)-β-d-Galf-(1→5)-β-d-Galf-(1→]_n_ [23].

Rugulosin (**18**) was isolated together with emodin and skyrin from *Hormonema dematioides* and showed activity against survival of budworm larvae while the other two anthraquinones were inactive [24].

*Aspergillus fumigatus*, which is responsible for a lung disease, produced a plethora of secondary metabolites belonging to different classes of natural compounds. Among them, emodin, 2-chloro-emodin (**21** and **22** Figure 3), and physcion (**23**, Figure 3) were also isolated. However, compound **23** appeared not to have a role in the fungal infection [25].

Physcion (**23**) was also isolated from the organic culture extract of the marine derived fungus *Microsporum* sp. and showed a cytotoxic effect on human cervical carcinoma HeLa cells and its apoptosis induction was deeply investigated. Physcion also caused the formation of reactive oxygen species (ROS) in the same cells [26].

Many *Drechslera* species, which are important pathogens on gramineous plants and their seeds, produced colored pigments [27]. The red pigment exudated from *Drechslera teres*, *D. graminea*, *D. tritici-repentis*, *D. phlei*, *D. dictyoides*, *D. avenae* was identified as the anthraquinone catenarin (**24**, Figure 3, Table 1) while that from *D. avenae* and *Bipolaris sorokiniana* were two other anthraquinones recognized as helminthosporin and cynodontin (**25** and **26**, Figure 3, Table 1). Catenarin (**24**) showed a total inhibition of *Bacillus subtilis* (Gram+) growth but had no effect on the Gram- bacterium *Ervinia carotova* but in part inhibited the mycelium growth of *D. teres* [28]. In particular, catanerin and emodin (**24** and **21**) were also found in kernels infected by *Pyrenophora tritici-repentis* (Died.) Drechs. (anamorph: *Drechslera tritici-repentis* (Died.) Shoem.), the causal agent of tan spot of wheat. Compound **24** caused the reddish discoloration with red smudge of kernels, while compound **21** indicated that *P. tritici-repentis* is a mycotoxigenic fungus. Compound **24** also induced non-specific leaf necrosis and appeared moderately active against some of the fungi associated with *P. tritici-repentis* suggesting its possible role in the life strategy of the pathogen [29].

Cytoskyrins A and B (**27** and **28**, Figure 3, Table 1) are two closely related bisanthraquinones obtained from large-scale cultures of an endophytic fungus, CR200 (*Cytospora* sp.), isolated from the branch of a *Conocarpus erecta* tree in the Guanacaste Conservation Area of Costa Rica. Previously, a substituted benzopryrone, named cytosporone, was isolated from the same fungus and showed antibiotic activity [30]. Cytoskyrin A showed strong BIA activity down to 12.5 ng in the standard assay while cytoskyrin B was inactive at the concentrations tested (<50 mg) [31]. The biochemical induction assay (BIA) measures the induction of the SOS response in bacteria and is used to identify compounds that inhibit DNA synthesis, either directly by inhibiting the DNA replication machinery or more often indirectly by modifying DNA [32,33,34]. BIA activity is highly dependent on the three-dimensional structure and not a general property of these polyphenolic compounds. In fact, close bisanthraquinones such as luteoskyrin (**29**, Figure 3) and rugulosin (**18**) are known to interact with DNA. The structural basis of these differences is not yet clear [31].

**Figure 4:**
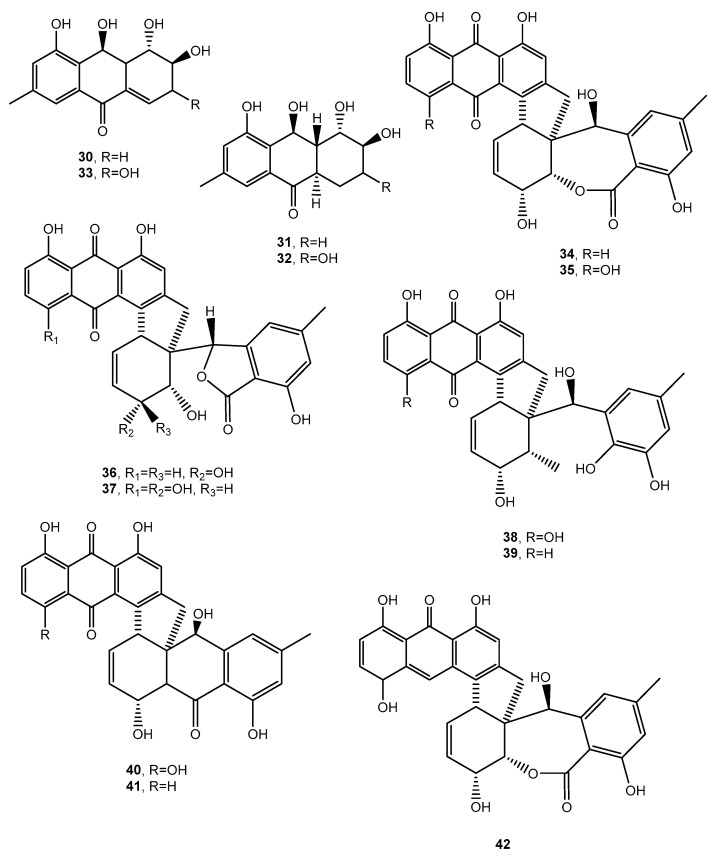
Bioactive anthraquinones and analogues produced by *Dendryphiella* sp., *Ramularia collo-cygni, Trichoderma harzianum and Ramularia uredinicola*.

Dendryols A–D (**30–33**, Figure 4, Table 1), four phytotoxic anthraquinones, were produced by *Dendryphiella* sp. [35], a fungus isolated from an infected sample of the paddy weed *Eleocharis kuroguwai* (Cyperaceae) in Japan [36]. The dendryols **30**–**33,** when tested for the phytotoxic activity by leaf-puncture assay on weeds (kuroguwai, barnyardgrass, and velvetleaf) and cultivated crops (rice, corn, and cowpea), showed toxicity only against barnyardgrass and the nercrotic area appeared to be dose-dependent. Compound **30** caused similar necrosis only on velvetleaf [35].

Rubellin A (**34**, Figure 4, Table 1) was isolated from the culture filtrates of *Ramularia collocygni*, the causal agent of leaf-spot disease of barley in central Europe [37]. From the same fungus rubellins B–F and 14-deydro rubellin D (**35**–**38**, Figure 4, Table 1) were also isolated. Biosynthetic studies carried out by the incorporation of both [1-^13^C]-acetate and [2-^13^C]-acetate into the rubellins demonstrated that such anthraquinone derivatives were biosynthesized via the polyketide pathway. Rubellin A (**34**) increased photodynamic oxygen activation [38], while rubellins B–E exhibited antibacterial activity, as well as light-dependent, antiproliferative and cytotoxic activity in a series of human tumor cell lines [39]. Closely related anthraquinones were isolated from the same fungus and from *Ramularia uredinicola* and identified as uridinetubellins I and II, and caeruleoramulin (**40**–**42**, Figure 4 and Table I). Both uredinorubellins (**40** and **41**) showed photodynamic activity comparable to rubellin D, whereas caeruleoramularin did not display such activity [40].

**Figure 5:**
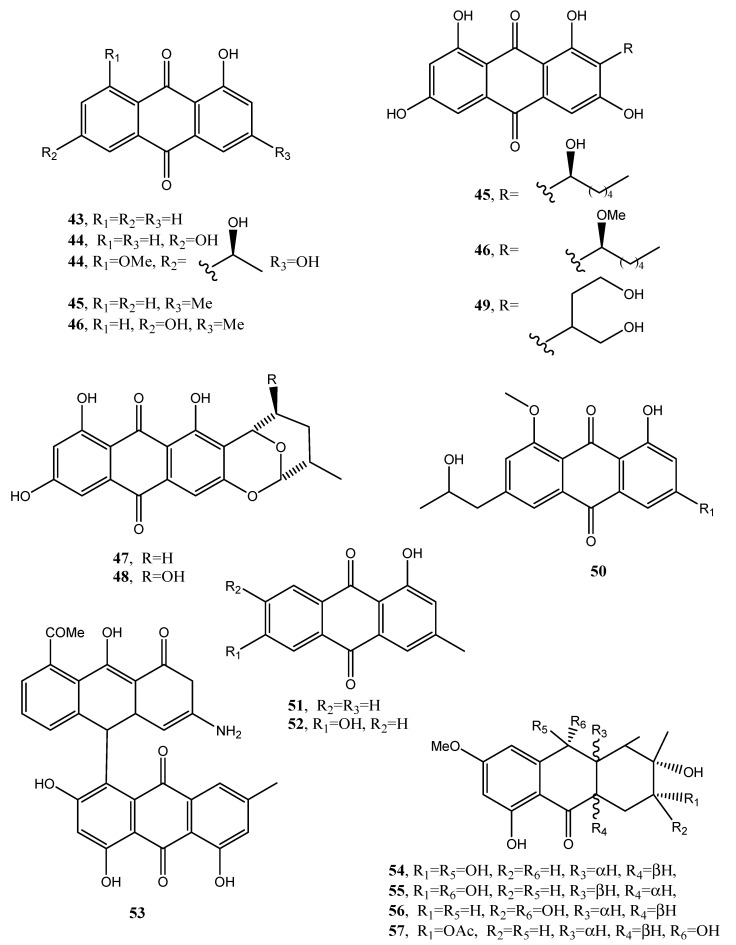
Bioactive anthraquinones and analogues produced by *Aspergillus versicolor, Phoma foevata, Coniothyrium* sp., *Phoma herbarum, Ascochyta lentis* and *Alternaria* sp.

1-Hydroxy-3-methyl-anthraquinone and 1,8-dihydroxy-3-methyl-anthraquinone (**43** and **44**, Figure 5, Table 1), were isolated together with other bioactive metabolites from *Trichoderma harzianum* in a study aimed to improve the production and application of novel biopesticides and biofertilizers and thus to help in the management of crop plant diseases. However, the two anthraquinones had no role in this activity [41].

Five anthraquinones named averantin, methyl-averantin, averufin, nidurufin, and versiconol (**45**–**49**, Figure 5, Table 1) were isolated from *Aspergillus versicolor,* a sponge-derived fungus. Methyl averatin and nidurufin (**45** and **48**) showed strong cytotoxicity against five human solid tumor cell lines (A-549, SK-OV-3, SK-MEL-2, XF-498, and HCT-15) with IC_50_ values in the range of 0.41–4.61 μg/mL.

Averatin and nidurufin (**45** and **48**) had antibacterial activity against several clinical isolates of Gram + strains with MIC values of 0.78–6.25 μg/mL [42]. Successively, a new anthraquinone, named isorhodoptilometrin-1-methyl ether (**50**, Figure 5, Table 1), was isolated together with the already known emodin, 1-methyl emodin, evariquinone, 7-hydroxyemodin, 6,8-methyl ether, siderin, arugosin C, and variculanol from the same fungus but obtained from the Red Sea green alga *Halimeda opuntia*. Compound **50** and siderin showed moderate antibacterial activity against *B. subtilis*, *B. cereus,* and *S. aureus* at 50 μg/disk, while, tested at 3 μg, only compound **50** exhibited mild solid tumor selectivity HEP-G2 with respect to the human normal cells (CFU) [43].

*Phoma* is a genus well known as producer of phytotoxin belonging to different classes of natural compounds including anthraquinones [44]. In fact, from *Phoma foevata*, the causal agent of gangrene in potatoes, several anthraquinones were isolated such as the already cited emodin and crysophanol, together with pachybasin and phomarin (**51** and **52**, Figure 5, Table 1) but no biological activity was reported [45]. Furthermore, a phytotoxic anthraquinone, which was identified as anhydropseudophlegmacin-9,10-quinone-3′-amino-8′-*O*-methyl ether (**53**, Figure 5, Table 1), was isolated from *Phoma herbarum* FGCC#54, a phytopathogenic fungus, investigated for herbicidal potential. Compound **53** showed phytotoxic activity against the prominent weeds of Central India [46].

**Figure 6:**
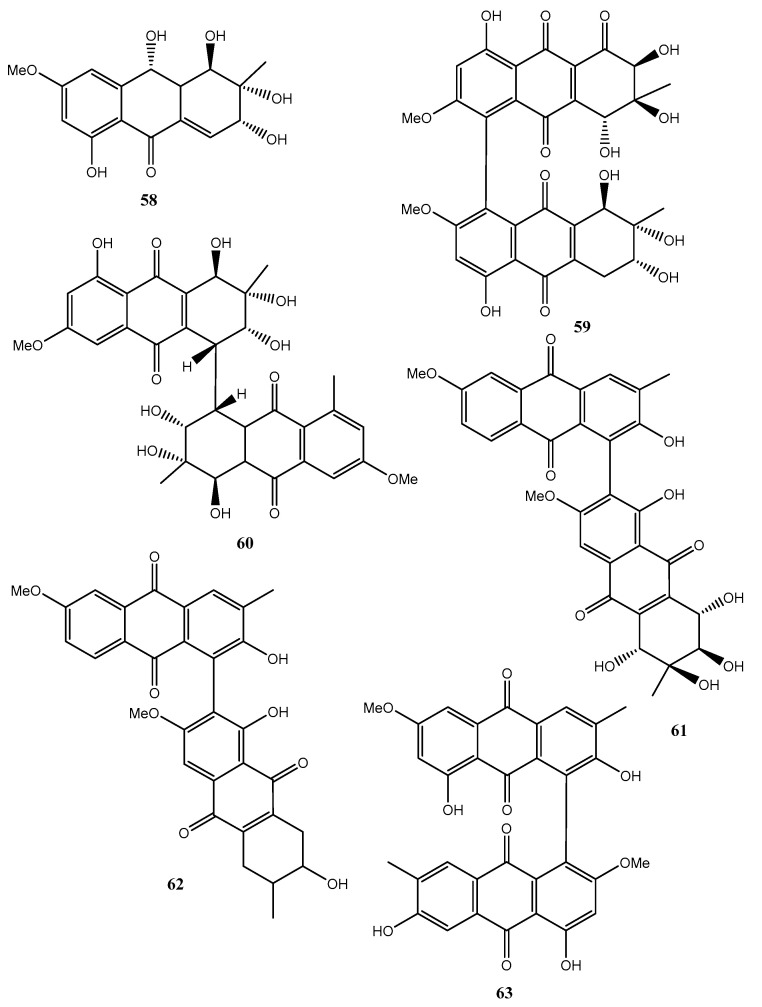
Bioactive anthraquinones and analogues produced by *Alternaria* sp.

Fungi belonging to the *Alternaria* genus are also well known as producers of a plethora of bioactive metabolites. In fact, five new hydroanthraquinone derivatives, named tetrahydroaltersolanols C–F (**54**–**57**, Figure 6, Table 1) and dihydroaltersolanol A (**58**, Figure 6, Table 1), and five new alterporriol-type anthranoid dimers, named alterporriols N−R (**59**–**63**, Figure 6), were isolated from the culture broth and the mycelia of *Alternaria* sp. ZJ-2008003.

The fungus also produced seven known analogues as tetrahydroaltersolanol B, altersolanol B, altersolanol C, altersolanol L, ampelanol, macrosporin (**8**, Figure 2) and alterporriol C. The fungus was isolated from *Sarcophyton* sp. soft coral collected from the South China Sea. All the compounds were assayed against the porcine reproductive and respiratory syndrome virus (PRRSV) and **54** and **62** showed antiviral activity with IC_50_ values of 65 and 39 μM, respectively. Compound **61** exhibited cytotoxic activity against PC-3 and HCT-116 cell lines, with IC_50_ values of 6.4 and 8.6 μM, respectively [47].

**Figure 7:**
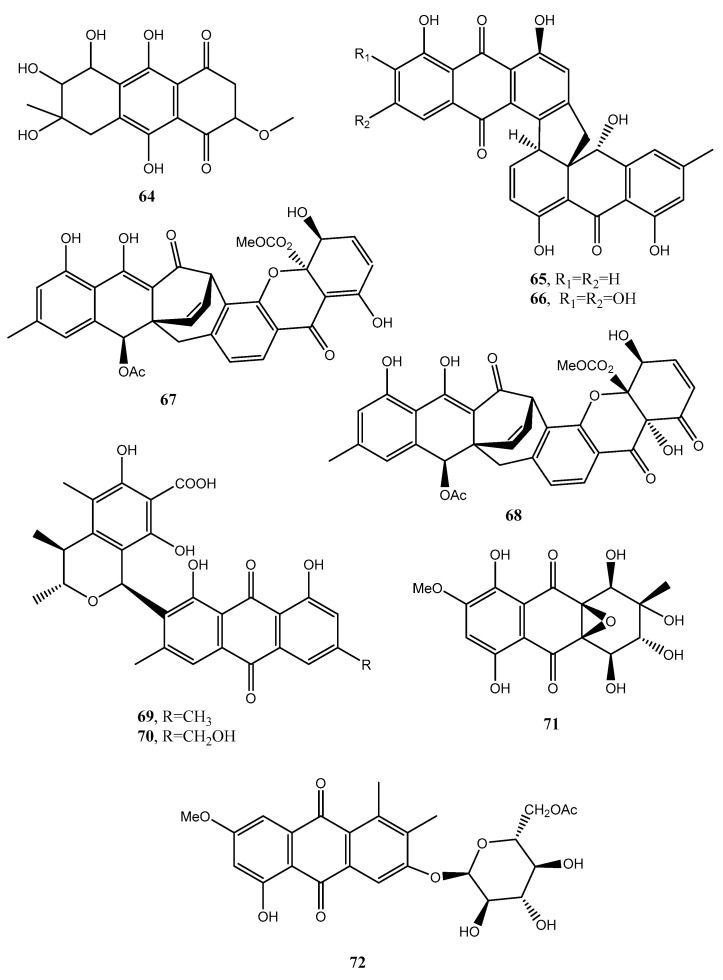
Bioactive anthraquinones and analogues produced by *Halorosellinia* sp., *Torrubiella* sp. BBC 28517, *Hypocreales* sp. MSX 17022, *Penicillium citrinum* PSU-F51 and *Stemphylium* sp. 33231.

Holoroquinone (**64**, Figure 7, Table 1), is an anthraquinone isolated from a marine-derived fungus *Halorosellinia* sp., which showed antitumor activity. Its biosynthesis was elucidated by incorporation of [2-^13^C]malonate and [1,2,3-^13^C]malonate followed by ^13^C-NMR investigation [48].

Two new dimeric anthraquinones with a rare chemical skeleton, named torrubiellins A and B (**65** and **66**, Figure 7, Table 1), were isolated from *Torrubiella* sp. BCC 28517 (family Clavicipitaceae) belonging to a genus of fungus that attacks spiders, scale-insects, and hoppers. Torrubiellin B (**66**) exhibited a broad range of biological activities including strong antimalarial (*Plasmodium falciparum*), antifungal (*Candida albicans*), antibacterial (*Bacillus cereus*) activities, and cytotoxicity to cancer cell lines. Its biological activity was always higher than that of torrubiellin A (**65**) [49].

Two new xanthone–anthraquinone heterodimers, named acremoxanthones C and D (**67** and **68**, Figure 7, Table 1), were isolated from an unidentified fungus of the *Hypocreales* order (MSX 17022). The fungus also produced the close and already known acremonidins A and C, benzophenone, and moniliphenone. All the metabolites showed moderate cytotoxic activity in vitro. In addition, acremoxanthone D (**68**), and acremonidins A and C exhibited moderate 20S proteasome inhibitory activity [50].

Two new anthraquinone citrinin derivatives, named penicillanthranins A and B (**69** and **70**, Figure 7, Table 1), were isolated together with 3*R*,4*S*-dihydrocitrinin from the mycelium extract of the sea fan-derived fungus *Penicillium citrinum* PSU-F51. Penicillanthranin A (**69**) showed moderate antibacterial activity against *Staphylococcus aureus* ATCC25923 with MIC values of 16 μg/mL and methicillin-resistant *S. aureus* SK1. Compounds **69** also exhibited mild cytotoxicity toward KB cells with IC_50_ values of 30 μg/mL [51]. Two new benzopyranones and one isochroman together with several known compounds as methyl 2-(2-acetyl-3,5-dihydroxy-4,6-dimethylphenyl)acetate, coniochaetone A, decarboxydihydrocitrinin, 1-acetonyl-7-carboxyl-6,8-dihydroxy-3,4,5-trimethylisochroman 6,8-dihydroxy-3,4,5,7-tetramethyl-3,4-dihydroisocoumarin, methyl 8-hydroxy-6-methylxanthone-1-carboxylate, sydowinin A, pinselin, conioxanthone A, chrysophanol, emodin, and ω-hydroxyemodin were isolated from the culture filtrates of the same fungus [51].

**Figure 8:**
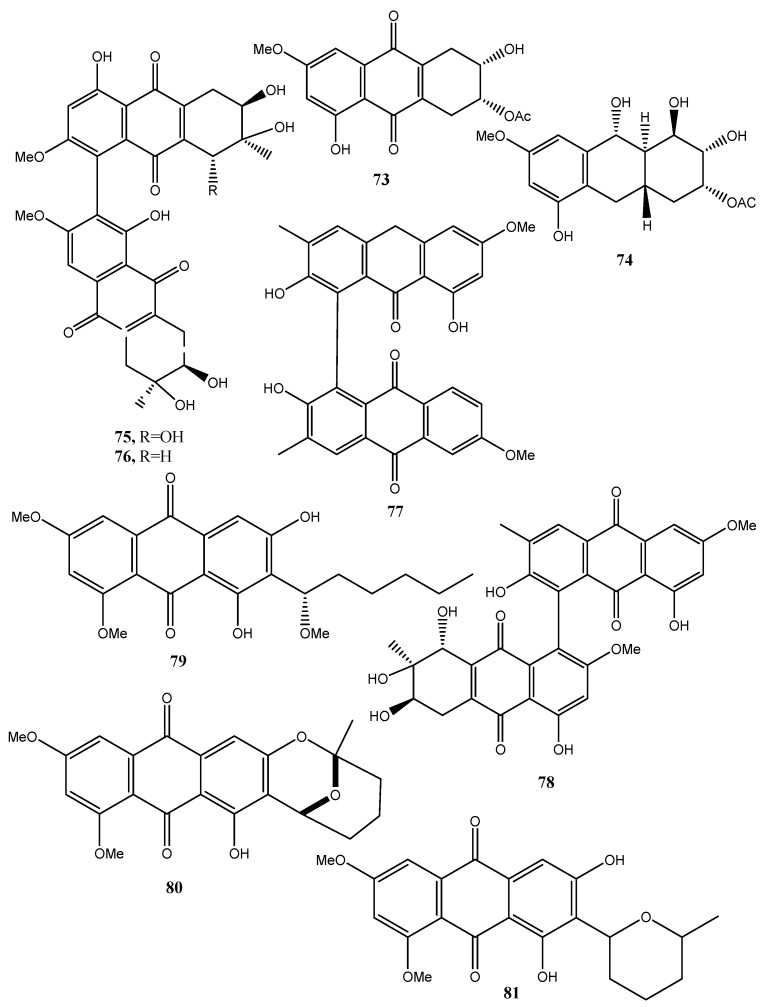
Bioactive antharaquinones and analogues produced by *Stemphylium* sp. 33231, *Penicillium purpurogenum*, the endophytic fungus ZSUH-36 and *A. versicolor.*

Four new anthraquinone derivatives, named auxarthrol C, macrosporin 2-*O*-(6′-acetyl)-α-d-glucopyranoside (**71** and **72**, Figure 7), 2-*O*-acetylaltersolanol B, and 2-*O*-acetylaltersolanol L (**73** and **74**, Figure 8), and four new alterporriol-type anthranoid dimers, named alterporriols T–W (**75**–**78**, Figure 8), were isolated along with 17 analogues from the rice culture of *Stemphylium* sp. 33,231 obtained from the mangrove *Bruguiera sexangula* var. *rhynchopetala* collected from the South China Sea [52]. The already known compounds were identified as dihydroaltersolanol A, macrosporin, macrosporin-7-*O*-sulfate, altersolanols A–C and L, ampelanol, tetrahydroaltersolanol B and alterporriols A–E, N, and Q. Compounds **78**, showed only weak antibacterial activity against *E. coli*, *S. aureus*, and *B. subtilis*, suggesting that anthraquinone derivatives showed better antibacterial activities than anthraquinone dimers in these assays. Compounds **78** also showed a moderate lethality, with an LD_50_ value of 10 μM when tested on brine shrimp lethality using *Artemia salina* [52].

**Figure 9:**
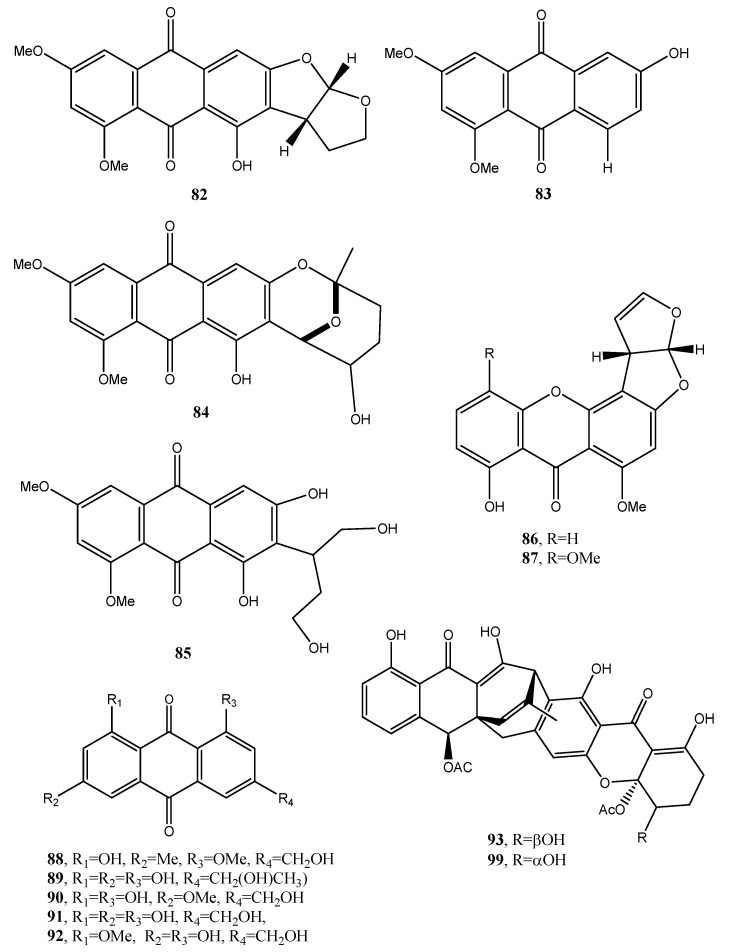
Bioactive anthraquinones and analogues produced by *P. purpurogenum*, the endophytic fungus ZSUH-36, *A. versicolor, Aspergillus* sp. YL-6, *Gliocladium* sp. T31 and *Engyodontium album.*

Eight known anthraquinone derivatives such as 6,8,10-tri-*O*-methyl averantin, 6,8-di-*O*-methyl averufnin, 6,8-di-*O*-methyl averufanin (**79**–**81**, Figure 8, Table 1) aversin, 1,3-dihydroxy-6,8-dimethoxy-9,10-anthraquinone, 6,8-di-*O*-methylnidurufin, 6,8-di-*O*-methyl versiconol and 5-methyoxysterigmatocystin (**82**–**86**, Figure 9, Table 1) were isolated from the extracts of *Penicillium purpurogenum* together with (*S*)-ornidazole. Only compounds **79** and **86** exhibited strong toxicity against brine shrimp (*Artemia salina*) at 10 mM, with lethality rates of 100% comparable to the positive control toosendanin. Compounds **79**, **82,** and **85** moderately inhibited the growth of *Botrytis cinerea*. Compound **82** showed moderate antifungal activity against *Gibberella saubinettii*, while compounds **84**, **85** and (*S*)-ornidazole exhibited phytotoxicity on radish seedlings at 100 mM [53]. 6,8,1′-Tri-*O*-methyl averantin (**79**), 6,8-di-*O*-methyl averufanin (**80**), and 6,8-di-*O*-methyl averufin (**81**), together with three known anthraquinones 1-*O*-methyl averantin, averufin (**9**), and versicolorin C were also produced by the endophytic fungus ZSUH-36 isolated from a mangrove collected from the South China Sea. At that time, only the unambiguous structure of **79**, being a new anthraquinone, was determined by advanced NMR spectra while no activity was described [54]. Previously, compounds **82** and **85,** together with the two xanthones 5-methoxysterigmatocystin and sterigmatocystin (**86** and **87**, Figure 9, Table 1), had been isolated from the same fungus and only the unambiguous structure of compound **85**, which at that time was a new anthraquinone, was determined by advanced NMR spectra [55]. Compounds **80**–**82** and **84** were previously isolated from the culture of *Aspergillus versicolor*, an endophytic fungus obtained from the marine brown alga *Sargassum thunbergii*.

Asperversin, A, 9ξ-*O*-2(2,3-dimethylbut-3-brevianamide Q, brevianamide K, brevianamide M, 6,8-5α,8α-epidioxyergosta-6,22-dien-3β-ol, ergosta-7,22-diene3β,5α,6β-triol, and 6β-methoxyergosta-7,22-diene-3β,5α-diol were isolated from the same fungus. Compound **81** exhibited antibacterial activity against *Escherichia coli* and *S. aureus*, and lethality against brine shrimp (*Artemia salina*) with an LC_50_ value of 0.5 μg/mL [56].

Two anthraquinones identified as questin and isorhodoptilometrin (**88** and **89**, Figure 9, Table 1) were produced together with chaetominine, (+)-alantrypinone, and 4-hydroxybenzaldehyde from the endophytic fungus *Aspergillus* sp. YL-6, isolated from the allelopathic plant *Pleioblastus amarus*. When all the metabolites were tested on wheat (*Triticum aestivum*) and radish (*Raphanus sativus*) at concentrations of 10 and 20 ppm, they inhibited the germination and growth of the two plant seeds completely. Among them (+)-alantrypinone, an indole-3-acetic acid (IAA), showed the best effects similar to that induced by glyphosate, a broad-spectrum systemic herbicide. Furthermore, questin (**88**), inhibited shoot and root elongation of wheat, always similar to glyphosate [57]. Questin was previously isolated together with another three anthraquinones, identified as fallacinol, citreorosein, and questinol (**90**–**92**, Figure 9, Table 1), and protocatechuic acid, (+)-catechin, 2,5-dimethyl-7-hydroxy chromone, 7-hydroxy-4-methoxy-5-methylcoumarin, torachrysone-8-*O*-d-glucoside, and 2-methoxy-6-acetyl-7-methyljuglone from the dried roots of *Polygonum cuspidatum*, a plant extensively used in Chinese and Japanese folk medicine [58]. Isorhodoptilometrin (**89**) was previously isolated together with secalonic acid D, emodin (**21**), and citreorosein (**91)**, from a marine lichen-derived fungus *Gliocladium* sp. T31 with secalinic acid D showing strong cytotoxic activity against human myeloid leukemia K562 cell line [59].

**Figure 10:**
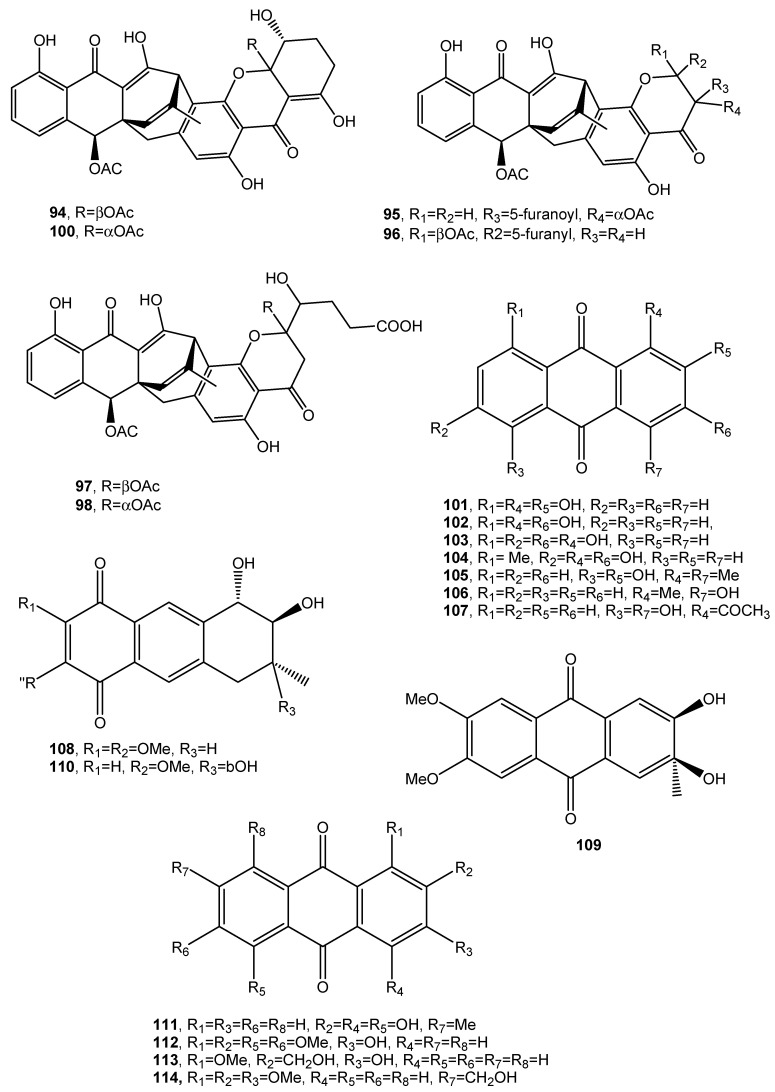
Bioactive anthraquinones and analogues produced by *Engyodontium album, Nigrospora* sp. and *Aspergillus tritici.*

Engyodontochones A (**93**, Figure 9, Table 1) and B–F (**94**–**97**, Figure 10, Table 1) together with two known polyketides as a betacolin-like metabolite (**99**, Figure 9, Table 1) and JBIR-99 (**100,** Figure 10, Table 1), all belonging to the anthraquinone–xanthone subgroup of polyketides, were isolated from mycelia and culture broth of *Engyodontium album* strain LF069. *E. album*, was found in soil extreme environments, plant debris, and in indoor environments on paper, textile, jute, and painted walls. It is a pathogenic fungus, inducing serious human diseases. Engyodontochones A–E (**93, 94**–**96**) and betacolin-like metabolite (**99**) exhibited inhibitory activity, 10-fold stronger than chloramphenicol, against methicillin resistant *S. aureus* (MRSA) [60].

Seven anthraquinone derivatives identified as 1,2,8-trihydroxyanthraquinone, 1,3,8-trihydroxyanthraquinone, 1,3,6-trihydroxy-8-methylanthraquinone, rheoemodin, aloesaponarin II, isozyganein, 1-acetyl-4,5-dihydroxy-anthraquinone (**101**–**107**, Figure 10, Table 1) were isolated together with *cis*-4-hydroxyscytalone, and cerebroside B from the culture filtrates of the endophytic fungus *Nigrospora* sp. *cis*-4-Hydroxyscytalone showed strong antibacterial activities against *E. coli* and *B. subtilis* with MIC values of 64 and 128 μg/mL, respectively. 1,3,8-Trihydroxyanthraquinone, 1,3,6-trihydroxy-8-methylanthraquinone, and aloesaponarin II (**102**, **103**, and **105**) exhibited inhibitory activity against *P. oryzae* with MIC values of 128 μg/mL, while 1,3,8-trihydroxyanthraquinone (**102**) showed moderate antifungal activity against *C. albicans* with MIC value of 128 μg/mL. Rheoemodin (**104)** exhibited weak antimicrobial activity only against *E. coli* with MIC values of 256 μg/mL [61]. Isozyganein (**106**) was also previously synthesized and showed weak antioxidative activity [62].

Aspetritones A and B (**108** and **109**, Figure 10, Table 1) were isolated from the culture of the coral-derived fungus *Aspergillus tritici* SP2-8-1, together with 4-methyl-candidusin A and fifteen known metabolites belonging to different classes of natural compounds as prenylcandidusin, candidusin, and terphenyllin derivatives and anthraquinones. Bostrocyn (**110**, Figure 10, Table 1) and other four anthraquinones (**111**–**114**, Figure 10, Table 1) were isolated. Aspetritone A (**108**) showed the most significant activity against methicillin-resistant strains of *S. aureus* in respect to that of the positive control chloramphenicol and exhibited strong cytotoxicity against human cancer cell lines HeLa, A549, and Hep G2 [63].

**Figure 11:**
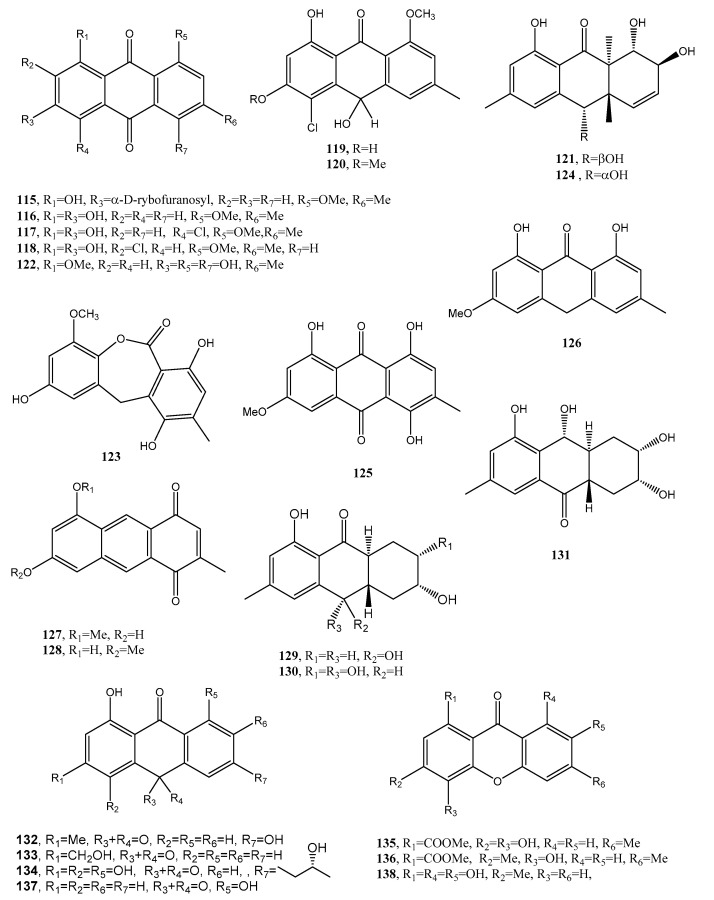
Bioactive anthraquinones and analogues produced by *Gaeumannomyces* sp., *Phialophora alba, Eurotium rubrum, A. glaucus, Coniothyrium* sp., *Penicillium* sp. OUCMDZ-4736, *Fusarium solani* and *Paraconiothyrium* sp.

A new anthraquinone glycoside derivative namely, 1-*O*-methyl-6-*O*-(α-d-ribofuranosyl)-emodin (**115**, Figure 11, Table 1), was produced together with two new resorcinol glycoside derivatives as resorcinol 2-butyl-5-pentyl-4-C-6-deoxy-β-d-gulopyranoside and resorcinol 2-butyl-5-pentyl-4-C-α-l-rhamnoside, named stemphols C and D, and eight already known compounds from the culture filtrates of the endophytic fungus *Gaeumannomyces* sp. isolated from the rhizome of a halophyte, *Phragmites communis*, in Suncheon bay, South Korea [64]. In particular, among the known compounds, 1-*O*-methylemodin (**116**, Figure 11, Table 1) was also identified. Compound **116** was first isolated together with 5-chloro-6,8-dihydroxy-1-methoxy-3-methylanthraquinone, 7-chloro-6,8-dihydroxy-1-methoxy-3-methylanthraquinone, 5-chloro-6,8,10-trihydroxy-1-methoxy-3-methyl-9(10*H*) anthracenone and 5-chloro-8,10-dihydroxy-l,6-dimethoxy-3-metbyl-9(10*H*)-anthracenone (**117**–**120**, Figure 11, Table 1), from *Phialophora alba*, a fungus that might protect the aspen from attack by other fungi [65]. Compounds **115** as well as the two glycosyl derivatives of resorcinol showed anti-inflammatory properties while 1-*O*-methylemodin (**116**) reduced NO production by LPS-treated cells by 43% and 31%, respectively, without inducing cell death [64].

Compound **115** also inhibited the growth of the tree decaying fungus *Phellinus tremulae* [66], the secretion of IL-625 [67], and of protein tyrosine phosphatase 1B [68].

Rubrumol (**121**, Figure 11, Table 1) was isolated together with four known analogues as emodin and catenarin (**21** and **24**), rubrocristin, 2-methyleurotinone, and conyothyrinone A (**122**–**124**, Figure 11, Table 1) from the solid culture of *Eurotium rubrum*, a fungus obtained from the salt-tolerance wild plant *Suaeda salsa* L. which was collected from ‘BoHai’ seaside. Among all the compounds, only **121** showed activity when tested on Topo I to relax supercoiled pBR322 DNA and it did not show cytotoxic activities against A549, MDA-MB-231, PANC-1, and HepG2 human cancer cell [69]. Rubrocristin (**122**) was previously isolated from the mycelia of *Aspergillus glaucus* together with physcion (**23**) emodin, and catenarin (**21** and **24**), questin (**88**), erythroglaucin, physcion-9-anthrone, viocristin, and isoviocristin (**125**–**128**, Figure 11, Table 1). Compounds **21**, **24**, **127,** and **128** showed antibacterial activity with minimal inhibitory concentrations ranging from 1–10 pg/mL, while compounds **21**, **24,** and **127** inhibited the incorporation of uridine and thymidine in Ehrlich ascites carcinoma cells while **24** and to a lesser extent compound **21** also inhibited in vitro DNA-dependent RNA polymerase from *Escherichia coli* [70]. Conyothyrinone A (**124**, Figure 11, Table 1) was also isolated from the culture of *Coniothyrium* sp. together with conyothyrinones B–D (**129**–**131**, Figure 11, Table 1) and the already known ones pachybasin and phomarin (**51** and **52**) as well as 1,7-dihydroxy-3-methyl-9,10-anthraquinone and 1-hydroxy-3-hydroxymethyl-9,10-anthraquinone (**132** and **133**, Figure 11, Table 1), an endophytic fungus isolated from *Salsola oppostifolia* from Gomera in the Canary islands. All these metabolites were tested for their antifungal, antibacterial, and algicidal properties. Compound **132** showing a strong antibacterial activity against *Bacillus magaterium* and *E. coli*. Phomarin and conyothyryrinone A (**52** and **124**) exhibited strong antifungal activity against *Mycrobotryum violaceum* and *B. cinerea*, while pachybasin (**51**), which is the main metabolite, showed weak activity against *B. megaterium* [71].

Three new anthraquinones, identified as (-)-2′*R*-1-hydroxyisorhodoptilometrin, methyl 3,4,8-trihydroxy-6-methyl-9-oxo-9*H*-xanthene-1-carboxylate, and methyl 6,8-dihydroxy-3-methyl-9-oxo-9*H*-xanthene-1-carboxylate (**134**–**136**, Figure 11, Table 1) were isolated from the culture filtrates, *Penicillium* sp. OUCMDZ-4736, on the effect of acid stress conditions. The fungus was isolated from the sediment around the roots of the mangrove (*Acanthus ilicifolius*). The new anthraquinones were isolated together with ten already known compounds including asterric acid, parietinic acid, endocrocin, and monochlorsulochrin [72]. Compound **134**, on comparison with the control lamivudine showed a stronger anti-hepatitis B virus inhibiting HBsAg and HBeAg secretion from HepG2.2.15 cells [73].

Danthron (**137**, Figure 11, Table 1), characterized as 1,8-dihydroxyanthraquinone, was produced as the main bioactive metabolite by the fungal endophyte *Paraconiothyrium* sp. isolated from *Zingiber officinale.* Compound **137** showed antifungal activity against clinical pathogens and against the phytopathogen *Pythium myriotylum*, which causes Pythium rot in ginger [74]. Danthron, also called as chrysazin, was used as a stimulant laxative in some countries and showed antibacterial, antifungal [75], and anticancer activities [76].

An aza-anthraquinone identified as bostrycoidin (**138**, Figure 11, Table 1) was produced together with anhydrofusarubin, fusarubin, 3-deoxyfusarubin, ergosterol, 3,5,9-trihydroxyergosta-7,22-diene-6-one, and 4-hydroxybenzaldehyde from large scale cultivation of the endophytic fungus *Fusarium solani* isolated from *Cassia alata* Linn. growing in Bangladesh.

The crude organic extract of the fungal culture filtrates showed cytotoxicity, using “Brine Shrimp Lethality Bioassay”, antimicrobial and antioxidant activity. Among the isolated metabolites, compound **138** appeared to be the most potent anticancer and antimicrobial metabolite [77].

**Figure 12:**
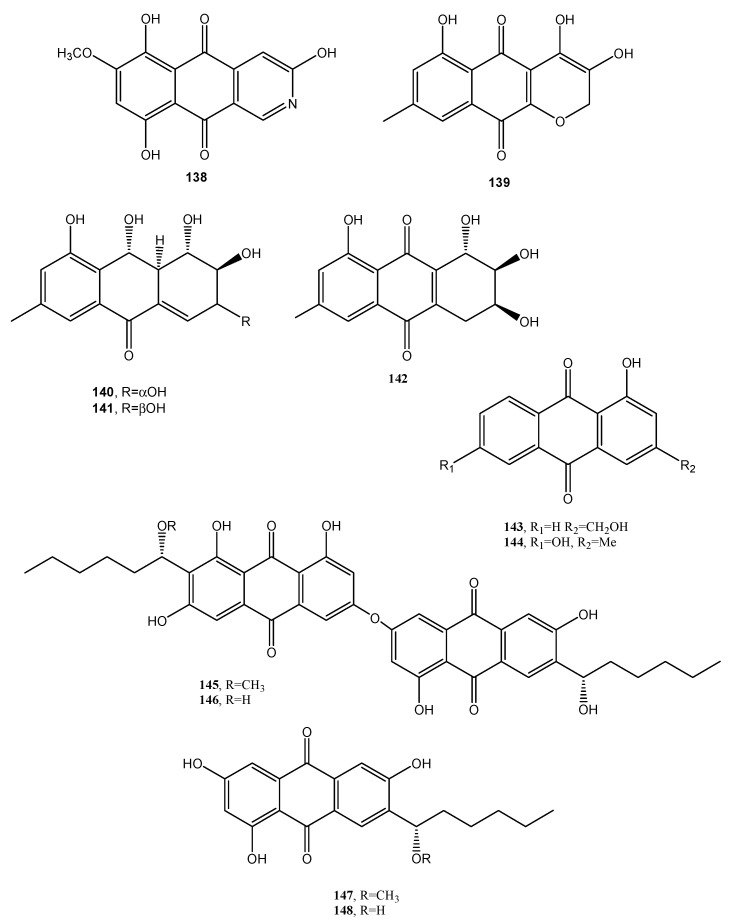
Bioactive anthraquinones and analogues produced by *Fusarium solani, Ascochyta lentis*, *A. versicolor* and *Aspergillus* sp. SCSIO F063.

Three new phytotoxic anthraquinones, named lentiquinones A–C (**139**–**141**, Figure 12, Table 1) were isolated from the culture filtrates of *Ascochyta lentis* inducing Ascochyta blight on lentil (*Lens culinaris* Medik.) [78]. Another new anthraquinone, named lentisone (**142** Figure 12, Table 1), together with the known pachybasin (**51**), tyrosol, and pseurotin A, was previously isolated from the same fungus [79]. Pachybasin (**51**) was also isolated, as the main metabolite, from the fungal mycelium of the same fungus together with other three known anthraquinones as ω-hydroxypachybasin (**143**, Figure 12, Table 1), 1,7-dihydroxy-3-methylanthracene-9,10-dione (**144,** Figure 12, Table 1), and phomarin (**52**) [78]. Tested by leaf-puncture on host and non-host plants, the three new anthraquinones (**139**–**141**) and lentisone (**142**) caused severe necrosis, with lentiquinone A being the most active. Compound **139** proved to be particularly active on cress (*Lepidium sativum*), in inhibiting root elongation. Furthermore, all the compounds reduced the content of chlorophyll in *Lemna minor*, with 1,7-dihyroxy-3-methylanthracene-9,10-dione (**144**) being the most active. The lentiquinones A–C and lenstisone had antibiotic properties [78].

**Figure 13:**
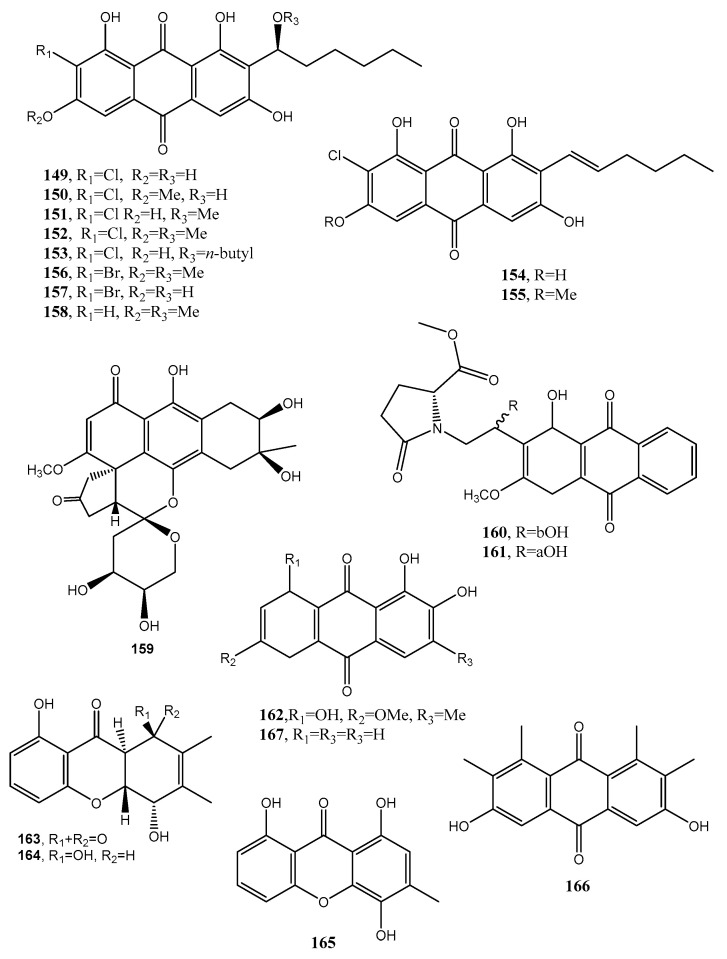
Bioactive anthraquinones and analogues produced by *Aspergillus* sp. SCSIO F063, *Alternaria tenuissima*, *Talaromyces funiculosus, Neofusicoccum luteum* and *Rubia tinctorum*.

Two anthraquinone dimers (**145** and **146**, Figure 12, Table 1) were produced together with another three known anthraquinones as 1′-*O*-methylaverantin and averantin (**147** and **148**, Figure 12, Table 1), averythrin (**7**), and two xanthones as stergmatocystin and variecoxanthone from the marine-derived fungus *Aspergillus versicolor*. Compounds **145** and **146,** showed selective antibacterial activity against *S. aureus,* while stergmatocystin exhibited moderate cytotoxicity against human cancer cell lines [15]. Averantin was also isolated from the marine-derived fungus *Aspergillus* sp. SCSIO F063 together with its seven related chlorinated anthraquinones as (1′*S*)-7-chloroaverantin, (1′*S*)-6-*O*-methyl-7-chloroaverantin, (1′*S*)-1′-*O*-methyl-7-chloroaverantin, (1′*S*)-6,1′-*O,O*-dimethyl-7-chloroaverantin, (1′*S*)-7-chloroaverantin-1′-butyl ether, 7-chloroaverythrin, and 6-*O*-methyl-7-chloroaverythrin (**149**–**155**, Figure 13, Table 1). Five known analogues, identified as 1′-*O*-methylaverantin, 6-*O*-methylaverantin, averantin-1′-butyl ether, and averythrin (**7**) were also isolated when the fungus was grown on sea salt-containing potato dextrose broth. When sodium bromide was added to the culture medium also two new brominated anthraquinones as (1′*S*)-6,1′-*O,O*-dimethyl-7-bromoaverantin and (1′*S*)-6-*O*-methyl-7-bromoaverantinone (**156** and **157**, Figure 13, Table 1) and a nonhalogenated anthraquinone, identified as (1′*S*)-6,1′-*O,O*-dimethylaverantin (**158**, Figure 13, Table 1) were extracted from fungal mycelia. Among all the compounds isolated only 6-*O*-methyl-7-chloroaveratin (**155)** displayed inhibition activity against three human tumor cell lines, SF-268, MCF-7, and NCI-H460, with IC_50_ values of 7.11, 6.64, and 7.42 μM, respectively [47].

A hydroanthraquinone with a hexacyclic spiro-fused ring system and two new anthraquinones with a 4,5-disubstituted butylaminolate unit, named anthrininones A–C (**159**–**161**, Figure 13, Table 1) were isolated from the deep-sea derived fungus *Alternaria tenuissima* DFFSCS013. They were isolated together with six known analogues including 6-*O*-methylalaternin (**162**, Figure 13, Table 1), 10,11- dihydroaltersolanol A, altersolanol L, ampelanol, (3*R*)-1-deoxyaustrocortilutein and altersolanol B. Compounds **159**–**162** showed strong inhibition activity against indoleamine 2,3-dioxygenase 1 (IDO1), and compounds **160**–**162** also had selective inhibition activity against different protein tyrosine phosphatase, while compound **159** stimulated intracellular calcium levels at a concentration of 10 μM [81].

Funiculosone (**163**, Figure 13, Table 1), a new substituted dihydroxanthene-1,9-dione, was isolated together with its two known analogues mangrovamide J and ravenelin (**164**–**165**, Figure 13, Table 1) from the culture filtrates of *Talaromyces funiculosus* (Trichocomaceae) an endolichenic fungus obtained from lichen thallus of *Diorygma hieroglyphicum* in India [82]. When assayed against *E. coli* and *S. aureus*, all the compounds displayed antibacterial activity with an IC_50_ range 23–104 μg/mL. Compound **163** also showed anticandidal activity against *Candida albicans* with an IC_50_ 35 μg/mL [82].

A new hexasubstituted anthraquinone, named neoanthraquinone (**166**, Figure 13, Table 1) was isolated from *Neofusicoccum luteum*, the causal agent of Botryosphaeria dieback in Australia. *N. luteum* produced also a new disubstituted furo-α-pyrone and a trisubstituted oxepi-2(7*H*)-one, named luteopyroxin and luteoxepinone respectively, together with the known (±)-nigrosporione, tyrosol, (*R*)-(−)-mellein and (3*R*,4*S*)-(−)- and (3*R*,4*R*)-(−)-4-hydroxymellein. Compound **166** caused severe shriveling and withering when assayed on grapevine leaves, while the other metabolites showed different degrees of toxicity [83].

## 3. Industrial Application of Anthraquinones

Since 1869 with the determination of the structure of alizarin (**167**, Figure 13, Table 1), a yellow anthraquinone, the main industrial application of this anthraquinone was its use as a dye in textile manufacturing [85]. Compound **167** was isolated for the first time from *Rubia tinctorum* [84]. Thus, over a span of 20 years many analogues with different functionalities were also prepared by synthesis to obtain different dyes such as red, blue, and green mordant. Successively, the first acidic anthraquinone dye used to color wool without pretreatment with mordants was reported. At the beginning of 1900 the sulfonation and nitration of anthraquinone opened up a new era for anthraquinone based dyes.

A new phase in this development occurred with the introduction of synthetic fibers, such as polyester, polyamide, and polyacrylonitrile fibers, with the substitution of anthraquinones with other dyes. The use of acid anthraquinone dyes increased with the discovery of the first fiber-reactive dyes. At the same time, the utilization of natural substances instead of synthetic ones, increased worldwide. This satisfy the request of environmentally friendly sustainable technologies. As reported in Section 1 fungi are a significant source of pigments as several genera can produce pigments in good amounts identified as anthraquinones or analogues. The production of anthraquinones by fungal fermentation had been developed for rapid and easy growth to produce pigments useful in various industrial applications [86]. The natural anthraquinones, as well as other natural pigments, have noteworthy less toxic effects than the synthetic dyes and are easily degradable avoiding the high environmental pollution. Thus, these anthraquinone based dyes are used in medical, textile coloring, food coloring, and cosmetic industries [84,86].

On this basis also plants have been largely used as a source of natural colored anthraquinones. In fact, screening of dyeing plants was carried out for their widespread use in previous centuries. Colorimetric analysis showed that the principal color was yellow-orange shades and could be attributed to flavonoids while the red colors were due to anthraquinones. Colors from plants that contain anthocyanins varied from blue-violet through to red. The nature of the support fibers (wool or cotton) plays an important role in the perceived colors [87].

At the same time several anthraquinone based dyes were synthesized for industrial applications as nitro derivatives useful also as dye intermediates. The reactions used were nitration methods for the preparation of 1- and 2-nitroanthraquinones and 1,5-, 1,8-, 1,6-, 1,7-, 1,8-, 2,6-, and 2,7-dinitroanthraquinone. These were also used to obtain their reduced analogues such as 1-amino-anthraquinone and 1,5-diaminoanthraquinones, both useful to produce vat dyes. Other methods were the preparation of 1-SO_3_H and 1-MeNH derivatives of anthraquinones useful for manufacturing of dyes for wool, acetate rayon, and polyamide fibers. Another method enables the preparation of leuco-1,4,5,8-tetrahydroxyanthraquinone useful for synthesis of acid and disperse dyes [88].

Recently water-repellent, self-cleaning and stain resistant textiles were obtained by developing anthraquinone reactive dyes which were covalently grafted onto cotton fabric surfaces obtaining bright colors with good wash-fastness properties and giving rise to breathable superhydrophobic textiles with self-cleaning properties [89].

The large number of textile dyes required a method for their classification which was based on the functional groups attached to the typical anthraquinone carbon skeleton. Thus, there are: anthraquinone, azo, phthalocyanine, sulfur, indigo, nitro, nitroso anthraquinone derivatives etc. taking into account their chemical structures. Another classification was based on the method of applying these dyes on an industrial scale, grouped as disperse, direct, acid, reactive, basic, vat dyes etc. [90].

The intensity of research focused on natural compounds has been growing over the past few decades. Anthraquinones have been most studied in China producing several publications which report different advanced extraction methods, analytical techniques, and industrial applications. These publications also describe the most used plants for anthraquinone content as Polygonaceae, Rubiaceae, and Fabaceae and report the best known anthraquinones: rhein aloe emodin, emodin, physcion, chrysophanol which are responsible for their numerous biological properties. Furthermore, the use of natural anthraquinones for industrial applications, has been described as an alternative to synthetic dyes to avoid some unwanted side effects [9].

However, the environmental contamination by wastewater containing dyes is today a severe problem to solve. The application of advanced oxidation processes (AOPs) to industrial wastewater has increased as well as an integrated approach for their biological and chemical treatment. The toxicity of the detergents and the dye have been determined in terms of effective concentration EC_50_ using mixed cultures of activated sludge as well as a pure culture of luminescent bacteria *Vibrio fischeri* NRRLB-11177. However, the dye was not degraded without AOP pretreatment, therefore the degree of its removal (decolorization) by the AOPs is an important preliminary stage of bio-sorption on activated sludge [91].

## 4. Conclusions

The sources, structures, and the biological activities of fungal bioactive anthraquinones were reported starting from 1966 to the present day. In the introduction the previous review published on this topic was also cited which did not however treat the topic extensively. The anthraquinones were chronologically described and in some cases their isolation and biological activity was investigated in depth. Furthermore, their industrial application in different fields, essentially as natural dyes, was also reported focusing on the comparison between natural and synthetic anthraquinone based dyes, their chemical derivatization and classification, and the advanced methods used in the treatment of the relative industrial wastewaters to avoid severe negative environmental pollution.

## Figures and Tables

**Table 1:** Anthraquinones and analogues produced by phytopathogenic and endophytic fungi.

Anthraquinone	Fungus	Biological Activity	Reference
Dothistromin (**1**, Figure 2)	*Dothistroma pini*	Phytotoxic	[13]
Bisdeoxydothistromin (**2**, Figure 2)	“ ^1^	No activity	[14]
Bisdeoxydehydrodothistromin (**3**, Figure 2)	“	“	“
6-Deoxyversicolorin C (**4**, Figure 2)	“	“	“
Averufin (**5**, Figure 2)	“	“	“
Nidurufin (**6**, Figure 2)	“	“	“
Averythrin (**7**, Figure 2)	*Dothistroma pini* *Aspergillus versicolor*	“	“ [15]
Macrosporin (**8**, Figure 2)	*Alternaria porri,* *Alternaria solani,* *Alternaria cucumerina,* *Diaporthe angelicae,* *Stemphyfium botryosum*	Phytotoxic	[16,17,20]
6-Methylxanthopurpurin 3 methyl eter (**9**, Figure 2)	*Alternaria bataticola* *Alternaria solani*	“	“
Alternasolanol A (**10**, Figure 2)	*D. angelicae*	”	[17]
Alternasolanol J (**11**, Figure 2)	“	“	“
Stemphylin (**12**, Figure 2)	*S. botryosum*	Phytotoxic and weak antitumor	[20]
Dactylariol (**13**, Figure 2)	“	Phytotoxic and in vitro anticancer	“
Stemphyperylenol (**14**, Figure 2)	*Stemphylium botryosum*	Weak phytotoxic	[21]
Alterporriol (**15**, Figure 3)	“	No activity	“
Stemphylenol A (**16**, Figure 3)	“	“	“
Stemphylenol B (**17**, Figure 3)	“	“	“
Rugulosin (**18**, Figure 3)	*Cryphonectria parasitica* *Hormonema dematioides*	Phytotoxic Insecticidal	[22,24]
Skyrin (**19**, Figure 3)	*”*	Phytotoxic	”
Crysophanol (**20**, Figure 3)	“	“	“
Emodin (**21**, Figure 3)	*C. parasitica*, *Pyrenophora tritici-repentis*, *Gliocladium* sp. T31, *Aspercgillus glaucus,* *H. dematioides,* *Aspergillus fumigatus,* *Phoma foevata*	Phytotoxic Mycotoxic Anticancer and inhibition in vitro DNA-dependent RNA polymerase	[22,24,25,29,45,59,70]
2-Chloroemodin (**22**, Figure 3)	*Aspergillus fumigatus*	No activity	[25]
Physcion (**23**, Figure 3)	“ *Microsporum* sp.	Anticancer	“ [26]
Catenarin (**24**, Figure 3)	*Drechslera teres, Drechslera graminea, Drechslera tritici-repentis, Drechslera phlei, Drechslera dictyoides, Drechslera avenae* *Aspergillus cristatus*	Antibiotic against Gram+ Phytotoxic Anticancer and inhibition in vitro DNA-dependent RNA polymerase	[28,29,70]
Helminthosporin (**25**, Figure 3)	*D. avenae,* *Bipolaris sorokiniana*	No activity	[28.29]
Cynodontin (**26**, Figure 3)	“	No activity	“
Cytoskyrin A (**27**, Figure 3)	*Cytospora* sp. CR200	BIA	[30,31]
Cytoskyrin B (**28**, Figure 3)	“	BIA	“
Luteoskyrin (**29**, Figure 3)	“	No activity	“
Dendryol A (**30**, Figure 4)	*Dendryphiella* sp.	Phytotoxic	[35]
Dendryol B (**31**, Figure 4)	“	“	“
Dendryol C (**32**, Figure 4)	“	“	“
Dendryol D (**33**, Figure 4)	“	“	“
Rubellin A (**34**, Figure 4)	*Ramularia collo-cygni*	Increased photodynamic oxygen activation	[37,38]
Rubellin B (**35**, Figure 4)		Phytotoxic, antibiotic, antiproliferative, and cytotoxic	[38,39]
Rubellin C (**36**, Figure 4)	“	“	“
Rubellin D (**37**, Figure 4)	“	“	“
Rubellin E (**38**, Figure 4)	“	“	“
Rubellin F (**39**, Figure 4)	*“*	No activity	“
Uridinetubellins I (**40**, Figure 4)	*Ramularia uredinicola* *Ramularia collo-cygni*	The photodynamic action toward three mammalian cell lines	[40]
Uridinetubellins II, (**41**, Figure 4)	“	“	“
Caeruleoramularin (**42**, Figure 4)	“	No activity	“
1-Hydroxy-3-methyl-anthraquinone (**43**, Figure 4)	*Trichoderma harzianum*	No activity	[41]
1,8-Dihydroxy-3-methyl-anthraquinone (**44**, Figure 4)	“	“	“
Averantin (**45**, Figure 5)	*Aspergillus versicolor*	Antibiotic, Cytotoxic	[42,45,48]
Methyl-averantin (**46**, Figure 5)	“	Cytotoxic	“
Averufin (**47**, Figure 5)	“	No activity	“
Nidurufin (**48**, Figure 5)	“	Antibiotic and cytotoxic	“
Versiconol (**49**, Figure 5)	“	No activity	“
Isorhodoptilometrin-1-methyl ether (**50**, Figure 5)	“	Antibiotic and mild anticancer	[43]
Pachybasin (**51**, Figure 5)	*P. foevata,* *Coniothyrium* sp., *Ascochyta lentis*	Weak antibiotic Antibiotic	[45,71,78]
Phomarin (**52**, Figure 5)	“	Antifungal activity Antibiotic	[45,71,78]
Anhydropseudophlegmacin-9,10-quinone-3′-amino-8′-*O*-methyl ether (**53**, Figure 5)	*Phoma herbarum*	Phytotoxic	[46]
Tetrahydroaltersolanol C (**54**, Figure 5)	*Alternaria* sp.	Antiviral	[47]
Tetrahydroaltersolanol D (**55**, Figure 5)	“	No activity	“
Tetrahydroaltersolanol E (**56**, Figure 5)	“	“	“
Tetrahydroaltersolanol F (**57**, Figure 5)	“	“	“
Dihydroaltersolanol A (**58**, Figure 6)	“	“	“
Alterporriol N (**59**, Figure 6)	“	“	“
Alterporriol O (**60**, Figure 6)	“	“	“
Alterporriol P (**61**, Figure 5)	“	Cytotoxic	“
Alterporriol Q (**62**, Figure 6)	“	Antiviral	“
Alterporriol R (**63**, Figure 6)	“	No activity	“
Holoroquinone (**64**, Figure 7)	*Halorosellinia* sp.	Antitumor	[48]
Torrubiellin A (**65**, Figure 7)	*Torrubiella* sp. BCC 28517	Moderate antimalarial, antifungal, antibacterial, cytotoxic	[49]
Torrubiellin A (**66**, Figure 7)	“	Antimalarial, antifungal, antibacterial, cytotoxic	“
Acremoxanthone C (**67**, Figure 7)	*Hypocreales* sp. MSX 17022	Moderate cytotoxic	[50]
Acremoxanthone D (**68**, Figure 7)	“	Moderate cytotoxic, and moderate 20S proteosome inhibition	“
Penicillanthranin A (**69**, Figure 7)	*Penicillium citrinum* PSU-F51	Moderate antibacterial and mild cytotoxic	[51]
Penicillanthranin B (**70**, Figure 7)	“	No activity	“
Auxarthrol C (**71**, Figure 7)	*Stemphylium* sp. 33231	“	[52]
Macrosporin 2-*O*-(6′-acetyl)-α-d-glucopyranoside (**72**, Figure 7)	“	“	“
2-*O*-Acetylaltersolanol B (**73**, Figure 8)	“	“	“
2-*O*-Acetylaltersolanol L (**74**, Figure 8)	“		“
Alterporriols T (**75** Figure 8)	“	“	“
Alterporriols U (**76** Figure 8)	“	“	“
Alterporriols V (**77** Figure 8)	“	“	“
Alterporriols W (**78** Figure 8)	“	Weak antibacterial and moderate zootoxic	“
6,8,1′-Tri-*O*-methyl averantin (**79**, Figure 8)	*Penicillium purpurogenum* Endophytic fungus ZSUH-36	Zootoxic and antifungal	[53,54]
6,8-Di-*O*-methyl averufnin (**80**, Figure 8)	*Penicillium purpurogenum* Endophytic fungus ZSUH-36 *Aspergillus versicolor*	No activity	[53,54,56]
6,8-Di-*O*-methyl averufanin (**81**, Figure 8)	“	Antibiotic and zootoxic	[53,54,56]
Aversin (**82**, Figure 9)	“	Antifungal	[53,55,56]
1,3-Dihydroxy-6,8-dimethoxy-9,10-anthraquinone (**83**, Figure 9)	*Penicillium purpurogenum*	No activity	[53]
6,8-Di-*O*-methylnidurufin (**84**, Figure 9)	*Penicillium purpurogenum* Endophytic fungus ZSUH-36 *Aspergillus versicolor*	Antifungal and phytotoxic	[53,54,56]
6,8-Di-*O*-methyl versiconol (**85**, Figure 9)	*Penicillium purpurogenum* Endophytic fungus ZSUH-36	Antifungal and phytotoxic	[53,55]
5-Methyoxysterigmatocystin (**86**, Figure 9)	“	Zootoxic	[53]
Sterigmatocystin (**87**, Figure 9)	*Penicillium purpurogenum*	No activity	[55]
Questin (**88**, Figure 9)	*Aspergillus* sp. YL-6, *Polygonum cuspidatum*	Allelopathy	[57,58]
Isorhodoptilometrin (**89**, Figure 9)	*Aspergillus* sp. YL-6, *Gliocladium* sp. T31	Alleopathy	[57,59]
Fallacinol (**90**, Figure 9)	*Polygonum cuspidatum*	No activity	[58]
Citreorosein (**91**, Figure 9)	*Polygonum cuspidatum* *Gliocladium* sp. T31	“	[58,59]
Questinol (**92**, Figure 9)	*Gliocladium* sp. T31	“	[58]
Engyodontochone A (**93**, Figure 9)	*Engyodontium album*	Antibiotic	[60]
Engyodontochone B (**94**, Figure 10)	“	No activity	“
Engyodontochone C (**95**, Figure 10)	“	Antibiotic	“
Engyodontochone D (**96**, Figure 10)	“	“	“
Engyodontochone E (**97**, Figure 10)	“	“	“
Engyodontochone F (**98**, Figure 10)	“	“	“
Betacolin-like compound (**99**, Figure 9)	“	Antibiotic	“
JBIR-99 (**100**, Figure 10)	“	No activity	“
1,2,8-Trihydroxyanthraquinone (**101**, Figure 10)	*Nigrospora sp.*	“	[61]
1,3,8-Trihydroxyanthraquinone (**102**, Figure 10)	“	Antifungal	“
1,3,6-trihydroxy-8-methylanthraquinone (**103**, Figure 10)	“	“	“
Rheoemodin (**104**, Figure 10	“	Antimicrobial	“
Aloesaponarin II (**105**, Figure 10)	“	Antifungal	“
Isozyganein (**106**, Figure 10)	“	Antioxidant	“
1-Acetyl-4,5-dihydroxy-anthraquinone (**107**, Figure 10)	“	No activity	
Aspetritone A (**108**, Figure 10)	*Aspergillus tritici*	Strong antibiotic and cytotoxic activity	[63]
Aspetritone B (**109**, Figure 10)	“	No activity	“
Bostrocyn (**110**, Figure 10)	“	“	“
Compound **111** (Figure 10)	“	“	“
Compound **112** (Figure 10)	“	“	“
Compound **113** (Figure 10)	“	“	“
Compound **114** (Figure 11)	“	“	“
1-*O*-methyl-6-*O*-(α-d-ribofuranosyl)-emodin (**115**, Figure 11)	*Gaeumannomyces* sp.	Anti-inflammatory Reduction of NO production by LPS-	[64]
1-*O*-Methylemodin (**116**, Figure 11)	*Gaeumannomyces* sp. *Phialophora alba*	Anti-inflammatory Reduction of NO production by LPS Growth inhibition of *Phellinus tremulae* Inhibition of the secretion of IL-625 Protein tyrosine phosphatase 1B inhibition	[64,65,66,67,68]
5-Chloro-6,8-dihydroxy-1-methoxy-3-methylanthraquinone (**117**, Figure 11)	*Phialophora alba*	No activity	[65]
7-Chloro-6,8-dihydroxy-1-methoxy-3-methylanthraquinone (**118**, Figure 11)	“	“	“
5-Chloro-6,8,10-trihydroxy-1-methoxy-3-methyl-9(10*H*) anthracenone (**119**, Figure 11)	“	“	“
5-chloro-8,10-dihydroxy-l,6-dimethoxy-3-metbyl-9(10*H*)-anthracenone (**120**, Figure 11)	“	“	“
Rubrumol (**121**, Figure 11)	*Eurotium rubrum*	Activity when tested on Topo I	[69]
Rubrocristin (**122**, Figure 11)	“ *Aspergillus* *glaucus*	No Activity	“
2-Methyleurotinone (**123**, Figure 11)	*Eurotium rubrum*	“	“
Conyothyrinone A (**124**, Figure 11)	*Eurotium rubrum* *Coniothyrium* sp.	Antifungal activity	[69,70,71]
Erythroglaucin (**125**, Figure 11)	*Aspergillus glaucus*	No activity	[70]
Physcion-9-anthrone (**126**, Figure 11)	“	“	“
Viocristin (**127** Figure 11)	“	Antibacterial activity Anticancer activity	“
Isoviocristin (**128**, Figure 11)	“	Antibacterial activity	“
Conyothyrinone B (**129**, Figure 11)	*Coniothyrium* sp.	Antimicrobial activity	[71]
Conyothyrinone C (**130**, Figure 11)	“	“	“
Conyothyrinone D (**131**, Figure 11)	“	“	“
1,7-Dihydroxy-3-methyl-9,10-anthraquinone (**132**, Figure 11)	“	Antimicrobial activity and strong antibacterial activity	“
1-Hydroxy-3-hydroxymethyl-9,10-anthraquinone (**133**, Figure 11)	“	Antimicrobial	“
(–)-2′*R*-1-hydroxyisorhodoptilometrin (**134**, Figure 11)	*Penicillium* sp. OUCMDZ-4736	Anti-hepatitis B virus	[72,73]
Methyl 3,4,8-trihydroxy-6-methyl-9-oxo-9*H*-xanthene-1-carboxylate. (**135**, Figure 11)	“	No activity	[72]
Methyl 6,8-dihydroxy-3-methyl-9-oxo-9*H*-xanthene-1-carboxylate (**136**, Figure 11)	“	“	“
Danthron (**137**, Figure 11)	*Paraconiothyrium* sp.	Antibacterial, antifungal and anticancer	[74]
Bostrycoidin (**138**, Figure 12)	*Fusarium solani*	Antimicrobial and anticancer	[77]
Lentiquinones A (**139**, Figure 12)	*Ascochyta lentis*	Phytotoxic and antimicrobial	[78]
Lentiquinones B (**140**, Figure 12)	*“*	“	“
Lentiquinones C (**141**, Figure 12)	“	“	“
Lentisone (**142**, Figure 12)	“	“	“
ω-Hydroxypachybasin (**143**, Figure 12	“	“	“
1,7-Dihydroxy-3-methylanthracene-9,10-dione (**144**, Figure 12)	“	“	“
Anthraquinone dimer (**145**, Figure 12)	*Aspergillus versicolor*	Selective antibacterial	[15]
Anthraquinone dimer (**146**, Figure 12)	“	“	“
1′-*O*-Methylaverantin (**147**, Figure 12)	“	No activity	“
Averantin (**148**, Figure 12)	“ *Aspergillus* sp. SCSIO F063	“	“ [80]
(1′*S*)-7-Chloroaverantin (**149**, Figure 13)	*Aspergillus* sp. SCSIO F063	“	[80]
(1′*S*)-6-*O*-Methyl-7-chloroaverantin (**150** Figure 13)		“	“
(1′*S*)-1′-*O*-Methyl-7-chloroaverantin (**151**, Figure 13)	“	“	“
(1′*S*)-6,1′-*O,O*-Dimethyl-7-chloroaverantin (**152**, Figure 13)	“	“	“
(1′*S*)-7-Chloroaverantin-1′-butyl ether (**153**, Figure 13)	“	“	“
7-Chloroaverythrin (**154**, Figure 13)	“	“	“
6-*O*-Methyl-7-chloroaverythrin (**155**, Figure 13)	“	Anticancer	“
(1′*S*)-6,1′-*O,O*-Dimethyl-7-bromoaverantin (**156**, Figure 13)	“	No activity	“
and (1′*S*)-6-*O*-Methyl-7-bromoaverantinone (**157**, Figure 13)	“	“	“
(1′*S*)-6,1′-*O,O*-Dimethylaverantin (**158**, Figure 13)	“	“	“
Anthrininone A (**159**, Figure 13)	*Alternaria tenuissima*	Inhibition activity against indoleamine 2,3-dioxygenase and stimulate intracellular calcium levels	[81]
Anthrininone B (**160**, Figure 13)	“	Inhibition activity against indoleamine 2,3-dioxygenase and against different protein tyrosine phosphatases	“
Anthrininone C (**161**, Figure 13)	“	“	“
6-*O*-Methylalaternin (**162**, Figure 12	“	“	“
Funiculosone (**163**, Figure 13)	*Talaromyces funiculosus*	Antimicrobial	[82]
Mangrovamide J (**164** Figure 13)	“	“	“
Ravenelin (**165** Figure 13)	“	“	“
Neoanthraquinone (**166**, Figure 13)	*Neofusicoccum luteum*	Phytotoxic	[83]
Alizarin (**167**, Figure 13)	*Rubia tinctorum*	Dye	[84]

^1^ This menas that the table cells contain the same concept.
